# Wnt3a disrupts GR-TEAD4-PPARγ2 positive circuits and cytoskeletal rearrangement in a β-catenin-dependent manner during early adipogenesis

**DOI:** 10.1038/s41419-018-1249-7

**Published:** 2019-01-08

**Authors:** Bongju Park, Soojeong Chang, Gwan-Jun Lee, Byeongsoo Kang, Jong Kyoung Kim, Hyunsung Park

**Affiliations:** 1 Department of Life Science, University of Seoul, Seoul, 02504 Republic of Korea; 2 SYSOFT R&D Center, Daegu, 42988 Republic of Korea; 3 Department of New Biology, DGIST, Daegu, 42988 Republic of Korea

## Abstract

Adipogenesis is a process which induces or represses many genes in a way to drive irreversible changes of cell phenotypes; lipid accumulation, round cell-shape, secreting many adipokines. As a master transcription factor (TF), PPARγ2 induces several target genes to orchestrate these adipogenic changes. Thus induction of *Pparg2* gene is tightly regulated by many adipogenic and also anti-adipogenic factors. Four hours after the treatment of adipogenic hormones, more than fifteen TFs including glucocorticoid receptor (GR), C/EBPβ and AP-1 cooperatively bind the promoter of *Pparg2* gene covering 400 bps, termed “hotspot”. In this study, we show that TEA domain family transcription factor (TEAD)4 reinforces occupancy of both GR and C/EBPβ on the hotspot of *Pparg2* during early adipogenesis. Our findings that TEAD4 requires GR for its expression and for the ability to bind its own promoter and the hotspot region of *Pparg2* gene indicate that GR is a common component of two positive circuits, which regulates the expression of both *Tead4* and *Pparg2*. Wnt3a disrupts these mutually related positive circuits by limiting the nuclear location of GR in a β-catenin dependent manner. The antagonistic effects of β-catenin extend to cytoskeletal remodeling during the early phase of adipogenesis. GR is necessary for the rearrangements of both cytoskeleton and chromatin of *Pparg2*, whereas Wnt3a inhibits both processes in a β-catenin-dependent manner. Our results suggest that hotspot formation during early adipogenesis is related to cytoskeletal remodeling, which is regulated by the antagonistic action of GR and β-catenin, and that Wnt3a reinforces β-catenin function.

## Introduction

The mouse 3T3-L1 cells have been widely used as an in vitro model system to investigate molecular mechanisms of adipogenesis^1^. The 3T3-L1 cells can be differentiated into mature adipocytes by two-day exposure to 3-isobutyl-1-methylxanthine (IBMX/M), dexamethasone (Dex/D), and insulin (I)^2–4^. Recently, genome wide analyses of DNase I hypersensitive regions revealed that after 4 h MDI treatment, approximately three times more DNA regions became accessible^5^. ChIP-seq analyses revealed that during such dynamic chromatin remodeling (within 4 h after MDI treatment), hundreds of DNA sequences covering 400 bps, termed “hotspots”, including the promoter of *Pparg2*, are cooperatively occupied with at least five TFs including C/EBPβ/δ, GR (also known as NR3C1), STAT5A, and other TFs. These early TFs recruit coregulators to induce chromatin remodeling and form early enhanceosomes at the hotspots^1,5,6^. Hotspots are enriched with enhancer histone marks, namely, H3K4me1, H3K4me2, and H3K27ac, suggesting that hotspots are key enhancers^1,7–9^. Similarly, intracellular and extracellular structures were also remodeled during adipogenesis when preadipocytes changed to round and lipid-laden adipocytes^4,10–12^. Within 24 h, MDI treatment rearranged the actin cytoskeleton from stress fibers to cortical structures in preadipocytes^13^. Thus, MDI dramatically rearranged both chromatin and cytoskeleton in this short period.

Canonical Wnt signaling represses adipogenesis, but enhances osteogenesis of mesenchymal stem cells^14,15^. 3T3-L1 preadipocytes secrete Wnt10b, which maintains preadipocytes in undifferentiated state via autocrine and paracrine signaling^16–19^. After binding to the frizzled receptor and coreceptor LRP5/6, the canonical Wnt ligand stabilizes β-catenin (also known as CTNNB1) by protecting it from the APC/AXIN/GSK-3β destruction complex. Stabilized β-catenin localizes in the nuclei and is recruited on Wnt target genes via protein-protein interactions with DNA-bound T-cell factor/lymphoid enhancer factor (TCF/LEF)^20–23^. As a coactivator, β-catenin increases the expression of TCF/LEF target genes such as *Axin2* and *Ccnd1*^24,25^. In addition, β-catenin maintains cytoskeletal integrity by interacting with membrane bound cadherins and α-catenin, which are connected to actin fibers^26–28^.

Previous studies on the anti-adipogenic effects of Wnt/β-catenin signaling focused on the inhibition of PPARγ activity by β-catenin^29–34^. However, how Wnt signaling prevents adipogenic hormones from de-repressing and activating the transcription of *Pparg2* during early adipogenesis (within 48 h after MDI treatment) remains unclear. Here, we showed for the first time that canonical Wnt signaling inhibits not only hotspot formation of *Pparg2*, but also cytoskeletal rearrangement in a β-catenin-dependent manner. These two events are regulated by the antagonistic actions of GR and β-catenin, and Wnt3a reinforces β-catenin function.

## Materials and methods

### Materials and antibodies

Insulin, dexamethasone, 3-isobutyl-1-methylxanthine, Hoechst33258, Oil Red-O and RU486 were purchased from Sigma-Aldrich (St. Louis, MO, USA). Recombinant mouse Wnt3a was purchased from R&D Systems (Minneapolis, MN, USA). Wnt3a-conditioned medium was obtained from confluent Wnt3a-expressing L929 cells^35^. Anti-PPARγ (E-8), C/EBPα (14AA), C/EBPβ (H-7), GR (M-20, G-5), p300 (C-20), CBP (A-22), KLF4 (H-180), c-Jun (H-79), and β-catenin (H-102) antibodies were purchased from Santa Cruz Biotechnology (Dallas, TX, USA). Anti-TAZ (M2-616) antibody was purchased from BD Biosciences (San Jose, CA, USA), anti-lamin A/C and anti-STAT5 antibodies from Cell Signaling Technology (Danvers, MA, USA), anti-FLAG (5A8E5), anti-Myc (2G8D5), and anti-HA (5E11D8) antibodies from GenScript (Piscataway, NJ, USA), anti-TEAD4 and anti-H3 antibodies from Abcam (Cambridge, MA, USA), anti-14-3-3γ (CG31-2B6) from Millipore (Billerica, MA, USA), and anti-β-actin (AC-15) from Sigma-Aldrich, fluorescent phalloidin conjugate from Invitrogen (Waltham, MA, USA). cDNAs of HA-GR or Myc-Tead4 was subcloned into the pLenti-vector. pBABE-3× FLAG-C/EBPβ and pCMV-3× FLAG vector have been described previously^36^. Primer sequences for qRT-PCR and ChIP-qPCR are summarized in Supplementary Table S1 and S2, respectively.

### Cells and adipocyte differentiation

3T3-L1 mouse preadipocytes (ATCC, Rockville, MD, USA) and mouse NIH-3T3 cells were maintained in DMEM containing 10% (v/v) bovine serum as described^35^. AmphoPack^TM^ 293 cells (BD Biosciences, San Jose, CA, USA), HEK-293T cells, C3H10T1/2 cells (ATCC, Rockville, MD, USA) and Wnt3a-L929 cells were maintained in DMEM containing 10% (v/v) FBS. For adipogenesis, post-confluent 3T3-L1 cells and C3H10T1/2 cells were exposed to the adipogenic cocktail (MDI) containing 2 μM D, 0.5 mM M, and 5 μg/ml I in DMEM supplemented with 10% FBS as described previously^35^. Lipid droplets were stained using Oil Red-O as described previously^37^.

### Generation of stable cell lines using retrovirus or lentivirus infection

C/β-NIH cells and EV-NIH cells were generated by infecting mouse NIH-3T3 cells with retroviruses encoding FLAG-tagged C/EBPβ and empty vector, respectively, using the pBABE retroviral vector system and HEK293-based packaging cells (AmphoPack^TM^ 293 cell line)^35^. GR-L1, Cβ-L1, or Tead4-L1 cells were generated by infecting mouse 3T3-L1 preadipocytes with lentiviruses expressing HA-tagged GR, FLAG-tagged C/EBPβ, or Myc-tagged TEAD4, respectively. GR/Cβ-L1 or EV/EV-L1 cells were generated by infecting Cβ-L1 and EV-L1 cells with lentiviruses encoding HA-tagged GR or empty vector, respectively, using the pWZL retroviral vector system. shTead4-L1, shβ-cat-L1, shGR-L1, and shCtrl-L1 cells were generated by infecting mouse 3T3-L1 cells with lentivirus encoding shRNA against mouse *Tead4* (5′-GCTGAAACACTTACCCGAGAA-3′), mouse *Ctnnb1* (5′-CCCAAGCCTTAGTAAACATAA-3′), mouse *Nr3c1* (encoding GR) (5′-TGAGATTCGAATGACTTATAT-3′) and control (5′-CCTAAGGTTAAGTCGCCCTCG-3′), respectively, using the pLKO.1 lentiviral vector system (Addgene, Cambridge, MA, USA).

### Chromatin immunoprecipitation (ChIP) and formaldehyde-assisted isolation of regulatory elements (FAIRE)

ChIP analyses were performed as described previously^38^. FAIRE analyses were performed using ChIP lysates (30 μg chromatin) as described previously^39^. Briefly, sonicated chromatin lysates were phase-separated by two rounds of phenol/chloroform extraction. Nucleosome-free DNA in the upper aqueous phase was obtained using ethanol precipitation. DNA was further treated with 10 μg RNase A and 20 μg proteinase K, and extracted using the QIAquick PCR purification kit (QIAGEN, Chatsworth, CA, USA). The isolated genomic DNAs were used for FAIRE-qPCR. The Ct value of a target gene in the isolated DNA sample of a ChIP or FAIRE experiment was normalized to the Ct value of the target gene in the input DNA (ΔCt = Ct _sample_−Ct _input_). The percentage of input indicates the value of 100 × 2^ΔCt^.

### Western blot analyses, nuclear extraction, and reporter assay

Western blot analyses were performed as described previously^35^. To obtain nuclear extracts, the cells were washed twice with ice-cold phosphate buffered saline (PBS), harvested, and then lysed with hypotonic buffer (20 mM Tris-HCl (pH 8.0), 10 mM NaCl, 0.2% NP-40, 10 mM β-glycerophosphate, 10 mM NaF, 1 mM Na_3_VO_4,_ and protease inhibitors) and incubated for 10 min on ice. The supernatant (cytosol extracts) was removed and the nuclear pellet was washed with hypotonic buffer and lysed with NETN buffer (20 mM Tris-HCl (pH 8.0), 140 mM NaCl, 0.5% NP-40, 10 mM β-glycerophosphate, 10 mM NaF, 1 mM Na_3_VO_4,_ and protease inhibitors), followed by 10 cycles of sonication (1 cycle; 30 s on, 30 s off). Nuclear extracts (supernatant) were obtained by centrifugation at 13,000 × *g* for 10 min at 4 °C. The reporter plasmids, C/EBP-Luc and GRE-Luc, contained the luciferase gene under the regulation of three copies of C/EBP binding sequences (GTTGCGCAAG) and one copy of glucocorticoid responsive element (GRE) (AGAACACTGTGTTCT), respectively. Reporter assays were performed using Lipofectamine reagent as described previously^40^. The pRL-TK plasmid encoding *Renilla* luciferase was cotransfected for normalizing transfection efficiency.

### Immunofluorescence of F-actin

The cellular F-actin was stained with fluorescent phalloidin conjugates (25 μΜ) for 40 min at room temperature prior to Hoechst staining. The stained cells were observed under a Zeiss LSM510 inverted confocal microscope according to the manufacturer’s instructions. F-actin structures in individual cells were categorized into three groups; S (stress) fiber, where F-actin stress fibers were observed both in the nucleus and cytoplasm; T (transition state) fiber, where F-actin stress fibers were observed in the cytoplasm but not in the nucleus; C (cortical structure), where F-actin stress fibers were observed neither in the nucleus nor in the cytoplasm, but were observed as cortical structures near the cellular membrane. Cells (13–48) in each treatment were observed and categorized into three groups.

### Statistical analysis

All quantitative measurements were performed in at least three independent experiments. Two-tailed unpaired Student’s *t*-tests were used to compare the data between controls and indicated experimental groups. *p-values < 0.05; **p-values < 0.01; ***p-values < 0.001 were considered statistically significant.

## Results

### Wnt3a inhibits early induction of Pparg2

**Fig. 1:**
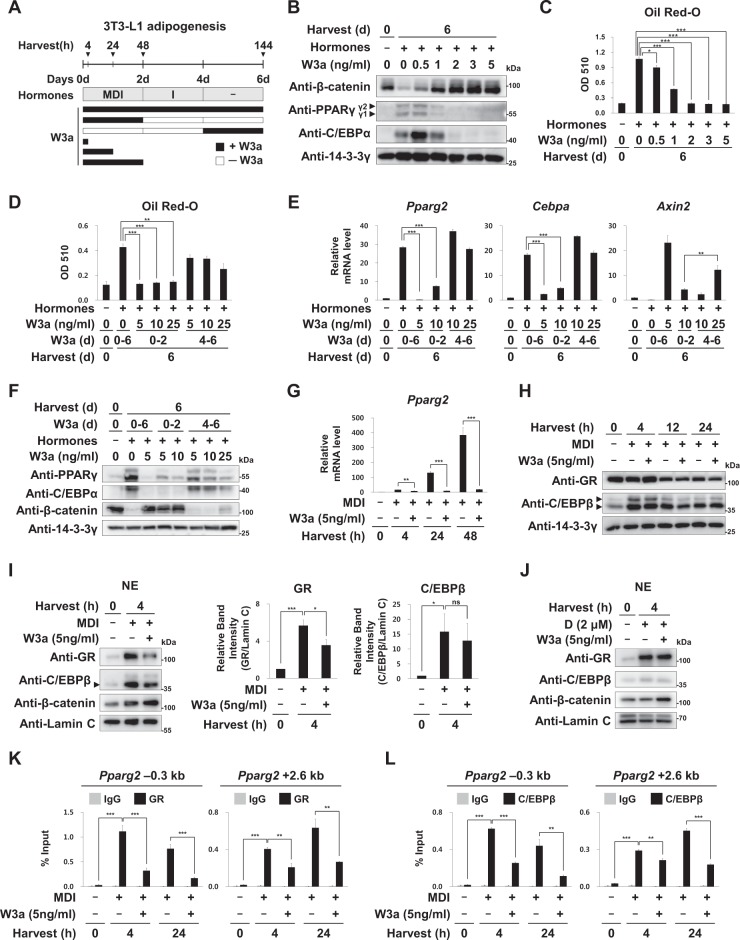
Effects of Wnt3a on early induction of *Pparg2*. **a** Post-confluent 3T3-L1 preadipocytes were induced to undergo adipogenesis by treatment with adipogenic hormones, IBMX (M), dexamethasone (D), and insulin (I) in the presence or absence of recombinant mouse Wnt3a (W3a). The treated cells were harvested at the indicated time points after induction. **b** Western blot analyses of 3T3-L1 cells using the indicated antibodies. 14-3-3γ was used as the loading control. Arrows indicate two PPARγ proteins (γ1, 54 kDa; γ2, 57 kDa). **c**, **d** Optical densities (510 nm) of Oil Red-O stained lipid in the 3T3-L1 cells at 6 days after the hormone treatment. The images of the Oil Red-O stained 3T3-L1 cells are shown in the Supplementary Fig. S1. **e** qRT-PCR analyses of *Pparg2*, *Cebpa*, and *Axin2* mRNA levels, which were normalized to 18S rRNA levels as described previously^35^. **f** Western blot analyses of 3T3-L1 cells using the indicated antibodies. **g** Relative mRNA levels of *Pparg2* to 18S rRNA levels. **h** Western blot analyses of 3T3-L1 cells using the indicated antibodies. Arrows indicate two active forms of C/EBPβ (36 and 38 kDa respectively). **i**, **j** Western analyses of nuclear extracts (NE) of 3T3-L1 cells treated with either MDI or Dex (D; 2 μM) in the presence or absence of W3a. Lamin C was used as the loading control for the nuclear proteins. The relative band intensities of GR, C/EBPβ (36 kDa, the lower band with a black arrowhead), and lamin C were determined using the ImageJ software from four independent western analyses (details in Supplementary Fig. S2A). **k**, **l** ChIP-qPCR analyses of GR or C/EBPβ occupancy on *Pparg2* (–0.3 kb or +2.6 kb from TSS) in 3T3-L1 cells. qPCR data show mean ± S.E. All data were repeated at least three independent same or similar experiments. *p < 0.05, **p < 0.01, and ***p < 0.001 by Students’ *t*-test; ns, not significant

We treated 3T3-L1 preadipocytes with adipogenic hormones (MDI) in the presence of recombinant Wnt3a (Fig. 1a). MDI induced the protein levels of both PPARγ and C/EBPα, but reduced β-catenin proteins. Two ng/ml of Wnt3a was sufficient to block the induction of both PPARγ and C/EBPα proteins and lipid accumulation but increased β-catenin proteins (Fig. 1b, c, and S1A). Although Wnt3a repressed both PPARγ1 and PPARγ2 proteins, PPARγ2 is major target for C/EBPβ and GR in response to MDI (Fig. S1C). Early temporal treatment of Wnt3a (5 ng/ml, for 0–2 days) was sufficient to block the early and later processes of adipogenesis, whereas more Wnt3a (>25 ng/ml) was required to block adipogenesis during the later processes (4–6 days time points) (Fig. 1d–g and S1B). Although late temporal treatment (for 4–6 days) of Wnt3a (25 ng/ml) increased the mRNA levels of *Axin2*, a Wnt target gene, it did not effectively reduce the mRNA and protein levels of *Pparg2* and *Cebpa* (Fig. 1e, f). These results confirmed the previous findings that the early period (within 0–2 days) is more sensitive to Wnt3a inhibition than the late period^17^.

Wnt3a significantly reduced the nuclear protein level of GR, but not C/EBPβ during early adipogenesis, although Wnt3a did not reduce the total protein levels of either GR or C/EBPβ (Fig. 1h, i and S2A). Interestingly, Wnt3a did not reduce GR protein level in the nuclei of the 3T3-L1 cells treated with only Dex, suggesting that Wnt3a specifically reduced nuclear GR only during early adipogenesis (Fig. 1j). Consistently, Wnt3a reduced GR binding at two major hotspots (−0.3 or +2.6 kb) located near the *Pparg2* transcription start site (TSS) (Fig. 1k). Although Wnt3a did not reduce C/EBPβ protein level, it inhibited the binding of C/EBPβ on the hotspots in *Pparg2* (Fig. 1l). We selected seven genes (*Acsl1, Hp, Tpcn2, Slc10a6, Krt13, Tsc22d3* and *Megf9*), the promoters (−5 to +1 kb from TSS) of which contain GR binding peaks, as identified in the ChIP-seq analyses^5^. We found that Wnt3a reduced GR occupancy in the promoter of all seven genes, which are also occupied by C/EBPβ; interestingly, Wnt3a also reduced C/EBPβ binding to these promoters (Fig. S2B). These results suggest that Wnt3a reduced GR and C/EBPβ occupancies on other MDI induced genes.

### Wnt3a disrupts cooperation between C/EBPβ and GR on the Pparg2 promoter

**Fig. 2:**
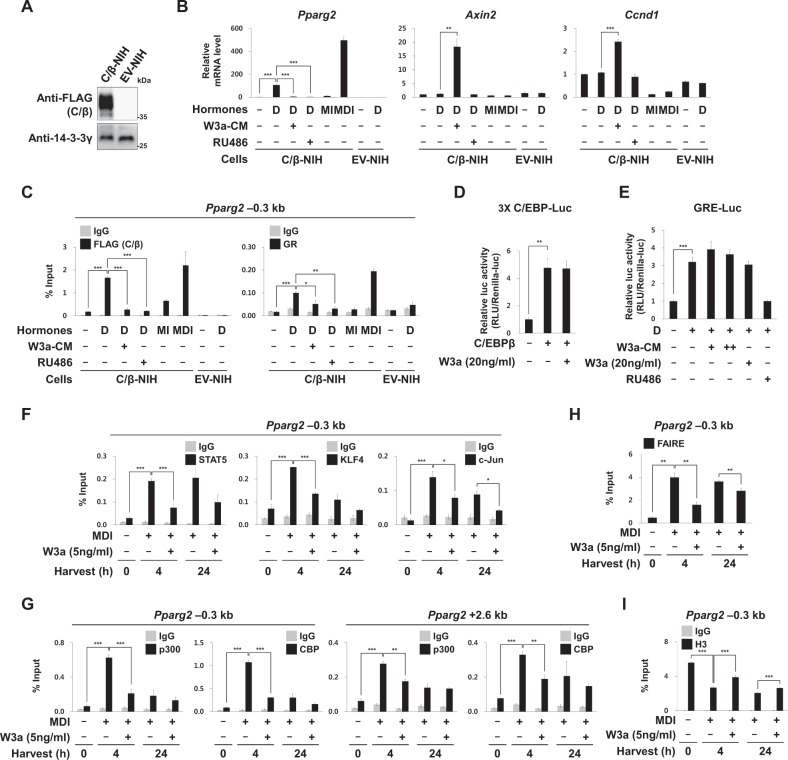
Effects of Wnt3a on cooperative binding of C/EBPβ and GR on *Pparg2*. **a**–**c** NIH-3T3 cells were infected with retrovirus encoding FLAG-tagged C/EBPβ (C/β-NIH cells) or empty vector as a control (EV-NIH cells). **a** Western blot analyses using anti-FLAG and 14-3-3γ antibodies. **b**, **c** Post-confluent C/β-NIH or EV-NIH cells were treated with the indicated adipogenic hormones, Wnt3a conditioned media (W3a-CM; 75%), or RU486 (10 μΜ) for 24 h. **b** qRT-PCR analyses of *Pparg2, Axin2*, and *Ccnd1* mRNA levels, which were normalized to 18S rRNA levels. **c** ChIP-qPCR analyses of FLAG (C/EBPβ) or GR occupancy on the –0.3 kb region from TSS of *Pparg2*. **d**, **e** Reporter analyses using C/EBP-Luc or GRE-Luc. The transfected 3T3-L1 cells were treated with Dex (D, 2 μM), W3a-CM (50 or 100%), recombinant mouse Wnt3a (W3a; 20 ng/ml), or RU486 (10 μM) for 24 h. **f**, **g** ChIP-qPCR analyses of STAT5, KLF4, c-Jun, p300, or CBP occupancy on *Pparg2* (–0.3 or +2.6 kb from TSS) in 3T3-L1 cells. **h** FAIRE-qPCR analyses on the –0.3 kb region of *Pparg2* in 3T3-L1 cells (details in Materials and methods). **i** ChIP-qPCR analyses of histone H3 on the –0.3 kb region from TSS of *Pparg2* in 3T3-L1 cells. qPCR data show mean ± S.E. All data were repeated at least three independent same or similar experiments. *p < 0.05, **p < 0.01, and ***p < 0.001 by Students’ *t*-test

To investigate whether the reduction in nuclear GR destabilized C/EBPβ binding to the *Pparg2* promoter, we generated C/β-NIH cells that ectopically express FLAG-tagged C/EBPβ (Fig. 2a). Dex alone (without IBMX) can induce *Pparg2* expression in C/β-NIH cells, as IBMX is required for the induction of C/EBPβ (Fig. 2b). Furthermore, the constitutively expressed C/EBPβ can bind the *Pparg2* promoter only when GR binds it in response to Dex treatment (Fig. 2c), and vice versa, GR binds to the *Pparg2* promoter only in C/β-NIH cells but not in EV-NIH cells. These results confirmed that C/EBPβ and GR interdependently bind the *Pparg2* promoter. Similar to Wnt3a, a GR antagonist, RU486, prevented not only GR but also C/EBPβ from binding to the *Pparg2* promoter (Fig. 2c).

In addition to the *Pparg2* promoter, C/EBPβ and GR can bind to different sets of their own target sequences. Analyses of the luciferase reporter gene show that Wnt3a does not inhibit C/EBPβ to induce the luciferase gene regulated by C/EBP binding sites (Fig. 2d). Similarly, RU486, but not Wnt3a, prevented Dex from inducing the luciferase gene, which was under the regulation of the glucocorticoid response element (GRE) (Fig. 2e). These results suggest that Wnt3a blocks neither C/EBPβ nor GR binding to their consensus motifs but disrupts the cooperativity between C/EBPβ and GR on the promoter of *Pparg2* by reducing GR level in the nuclei during early adipogenesis. We investigated whether Wnt3a also disrupted the cooperative binding of other hotspot TFs. We found that Wnt3a also reduced binding of STAT5, KLF4, and c-Jun to *Pparg2* (Fig. 2f). The cooperative bindings of several TFs significantly increased the recruitment of enhancer-associated coregulators, p300 and CBP, to form enhanceosomes^8,41^ and increased the accessibility of the chromatin structure of *Pparg2*^1^. As expected, Wnt3a blocked the recruitment of p300 and CBP on *Pparg2* in the MDI-treated cells (Fig. 2g). FAIRE (that detects nucleosome-depleted regions in the genome) and H3 ChIP analyses showed that MDI treatment reduced histone occupancy at the *Pparg2* promoter, but not in the presence of Wnt3a, suggesting that Wnt3a inhibits MDI-induced chromatin opening of *Pparg2* (Fig. 2h, i).

### Wnt3a prevents GR from inducing Tead4, a novel hotspot TF of Pparg2

**Fig. 3:**
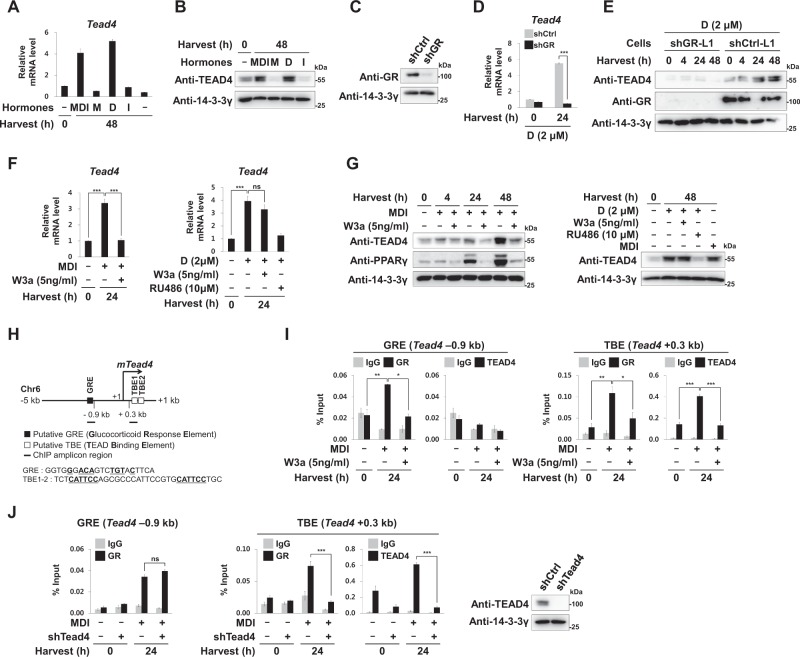
GR-induced TEAD4. **a** qRT-PCR analyses of *Tead4* mRNA of 3T3-L1 cells treated with adipogenic hormones as indicated. **b** Western blot analyses of 3T3-L1 cells using anti-TEAD4 and 14-3-3γ antibodies. **c** 3T3-L1 preadipocytes were infected with lentiviruses encoding shRNAs against mouse *GR* (shGR-L1 cells) or control shRNA (shCtrl-L1 cells). Western blot analyses showing GR protein levels in the shCtrl-L1 cells or the shGR-L1 cells. **d**, **e** The shCtrl-L1 cells or the shGR-L1 cells were treated with Dex (D, 2 μΜ) for the indicated time points. **d** Relative mRNA levels of *Tead4* to 18S rRNA levels. **e** Western analyses showing TEAD4 protein levels. **f**, **g** The 3T3-L1 cells were treated with MDI or Dex in the presence or absence of W3a or RU486 for the indicated time points. **f** Relative mRNA levels of *Tead4* to 18S rRNA levels. **g** Western analyses showing TEAD4 protein levels. **h** Indication of a putative GRE site (closed box) and TBE sites (open boxes) on *Tead4* (–5 to +1 kb) at chromosome 6. Black bars indicate ChIP amplicon regions (–0.9 and +0.3 kb from TSS of *Tead4* gene). **i**, **j** ChIP-qPCR analyses of GR or TEAD4 occupancy on *Tead4* (–0.9 or +0.3 kb from TSS) in 3T3-L1, shCtrl-L1, or shTead4-L1 cells. Western analyses showing TEAD4 protein levels of shCtrl-L1 cells or shTead4-L1 cells (right panel). qPCR data show mean ± SE. All data were repeated at least three independent same or similar experiments. *p < 0.05, ** *p* < 0.01, and *** *p* < 0.001 by Students’ *t*-test; ns, not significant

Starick et al. showed that TEA domain transcription factors (TEADs) reinforced GR binding to a subset of GR target genes as a heterodimer binding partner of GR^42^. For the first time, we found that Dex is responsible for inducing the mRNA and protein levels of *Tead4* in a GR-dependent manner (Fig. 3a–e and S3A to S3C). Interestingly, Wnt3a completely blocked MDI from inducing the mRNA and protein levels of *Tead4*, but did not block Dex’s ability to induce *Tead4* (Fig. 3f, g). These results are consistent with the findings that Wnt3a inhibits GR function in MDI-treated cells with higher sensitivity than in Dex-treated 3T3-L1 cells (Fig. 1i, j). ChIP revealed that both GR and TEAD4 occupied their putative binding sites in *Tead4* after MDI treatment (Fig. 3h, i). Interestingly, we found that GR bound to not only GRE (−0.9 kb) but also to TEAD binding elements (TBE) (+0.3 kb) in *Tead4* (Fig. 3h, i), and that knockdown of Tead4 reduced GR binding on the TBE of *Tead4* (Fig. 3j). These results suggest that GR and TEAD4 can cooperatively bind to the TBE of *Tead4*, and that *Tead4* is the target of TEAD4 itself as well as GR. Wnt3a prevented both GR and TEAD4 from binding to the GRE and the TBE of *Tead4* during early adipogenesis (Fig. 3i).

**Fig. 4:**
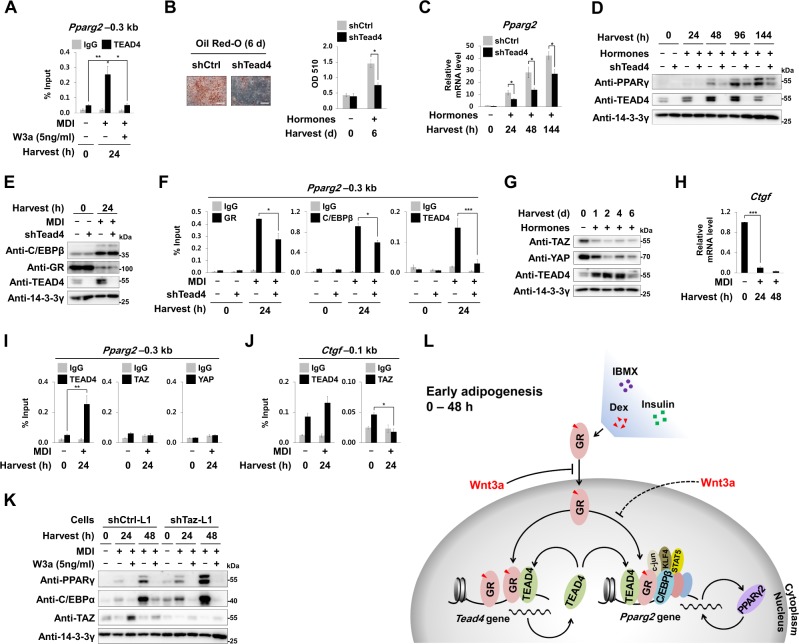
TEAD4 as a novel hotspot TF for *Pparg2* induction. **a** ChIP-qPCR analyses of TEAD4 occupancy on the –0.3 kb region from TSS of *Pparg2* in 3T3-L1 cells. **b**–**f** The shCtrl-L1 cells or the shTead4-L1 cells were induced to undergo adipogenesis by treating with adipogenic hormones for the indicated time points as described in Fig. 1a. **b** Images and optical densities (510 nm) of Oil Red-O stained lipid. Scale bars, 200 μm. **c** Relative mRNA levels of *Pparg2* to 18S rRNA levels. **d**, **e** Western blot analyses using the indicated antibodies. **f** ChIP-qPCR analyses of GR, C/EBPβ, or TEAD4 occupancy on the –0.3 kb region from TSS of *Pparg2*. **g**–**j** 3T3-L1 cells were treated with MDI for the indicated time points. **g** Western blot analyses of 3T3-L1 cells using the indicated antibodies. **h** Relative mRNA levels of *Ctgf* to 18S rRNA levels. **i,** **j** ChIP-qPCR analyses of TEAD4, TAZ, or YAP occupancy on the –0.3 kb region from TSS of *Pparg2* and the –0.1 kb region from TSS of *Ctgf*. **k** Western blot analyses using the indicated antibodies in the shCtrl-L1 cells and shTaz-L1 cells. **l** Schematic diagram showing GR-TEAD4-PPARγ2 positive circuits during early adipogenesis. Wnt3a disrupted two mutually related positive circuits by limiting the nuclear localization of GR. qPCR data show mean ± SE. All data were repeated at least three independent same or similar experiments. *p < 0.05, **p < 0.01, and ***p < 0.001 by Students’ *t*-test

Furthermore, TEAD4 also bound to the *Pparg2* promoter during early adipogenesis. We found that Tead4 knockdown reduced *Pparg2* induction and adipogenesis (Fig. 4a–d). Interestingly, Tead4 knockdown did not reduce GR and C/EBPβ protein levels but reduced their binding to the *Pparg2* promoter (Fig. 4e, f), suggesting that TEAD4 reinforced GR binding not only to the *Tead4* promoter, but also to the *Pparg2* promoter, for strong induction of both *Pparg2* and *Tead4*. Although TEADs are major transcription factors that convey the Hippo signal by recruiting their coactivators TAZ/YAP, TEAD4 recruited neither YAP nor TAZ on *Pparg2* (Fig. 4g–j). We also found that TEAD4 binds on the promoter of other MDI-induced genes, but do not recruit TAZ or YAP (Fig. S4A). Furthermore, TAZ knockdown did not block Wnt3a inhibitory effects suggesting that TAZ is not essential for the anti-adipogenic function of Wnt3a (Fig. 4k and Fig. S4B to S4E). We showed for the first time that TEAD4 and TAZ/YAP oppositely regulate the expression of *Pparg2*. These findings indicated that TEAD4 and GR form a positive circuit for induction of both *Tead4* and *Pparg2*. Thus, Wnt3a disrupted two mutually related positive circuits by blocking GR binding to the promoters of *Tead4* and *Pparg2* (Fig. 4l).

### Overexpression of GR is sufficient for blocking the inhibitory effects of Wnt3a

**Fig. 5:**
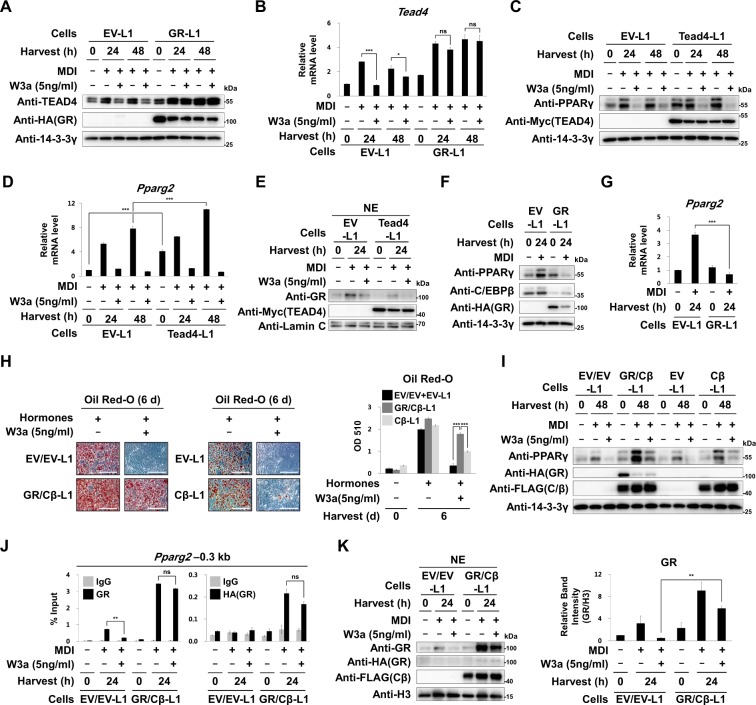
Effects of GR and C/EBPβ overexpression. **a**–**g** 3T3-L1 preadipocytes were infected with lentiviruses encoding HA-tagged GR (GR-L1), Myc-tagged TEAD4 (Tead4-L1) or empty vector as a control (EV-L1 cells). These cells were treated with MDI in the presence or absence of W3a for the indicated time points. **a** Western analyses of TEAD4 and HA-tagged GR protein. **b** Relative levels of *Tead4* mRNA to 18S rRNA. **c** Western analyses of PPARγ and Myc-tagged TEAD4. **d** Relative levels of *Pparg2* mRNA to 18S rRNA. **e** Western analyses of nuclear extracts (NE) of the EV-L1 cells or the Tead4-L1 cells treated with MDI in the presence or absence of W3a for 24 h using the indicated antibodies. Lamin C was used as the loading control for the nuclear proteins. **f** Western blot analyses using the indicated antibodies. **g** Relative levels of *Pparg2* mRNA to 18S rRNA. **h**–**k** 3T3-L1 preadipocytes were infected with lentiviruses encoding FLAG-tagged C/EBPβ (Cβ-L1) or empty vector as a control (EV-L1 cells). Cβ-L1 cells and EV-L1 cells were further infected with retrovirus encoding HA-tagged GR (GR/Cβ-L1) or empty vector (EV/EV-L1 cells), respectively. These cells were treated with MDI in the presence or absence of W3a for the indicated time points. **h** Images and optical densities (510 nm) of Oil Red-O stained lipid. Scale bars, 200 μm. **i** Western analyses using the indicated antibodies. **j** ChIP-qPCR analyses of GR or HA-tagged GR occupancy on the –0.3 kb region from TSS of *Pparg2*. *p* = 0.012 for GR and *p* = 0.085 for HA (GR). **k** Western analyses of nuclear extracts (NE) of the EV/EV-L1 cells or GR/Cβ-L1 cells treated with MDI in the presence or absence of W3a. H3 was used as the loading control for nuclear proteins. The relative band intensities of GR and H3 were determined using the ImageJ software from two independent western blot analyses. qPCR data show mean ± SE. All data were repeated at least three independent same or similar experiments. *p < 0.05, **p < 0.01, and ***p < 0.001 by Students’ *t*-test; ns, not significant

We investigated whether overexpression of ectopic GR is sufficient to resume the MDI-mediated induction of *Tead4* in Wnt3a-treated 3T3-L1 cells. We found that GR overexpression prevented Wnt3a from reducing the mRNA and protein levels of *Tead4*. These results confirmed that Wnt3a reduced the expression of *Tead4* by limiting the GR nuclear protein level during early adipogenesis (Figs. 1i,  5a, b). Overexpression of ectopic TEAD4 further increased the mRNA and protein levels of *Pparg2* in MDI-treated as well as untreated 3T3-L1 cells, confirming that TEAD4 is a positive regulator of *Pparg2* (Fig. 5c, d). However, in TEAD4 overexpressing cells, Wnt3a still limited GR protein level in the nuclei and reduced MDI-mediated induction of *Pparg2*, suggesting that TEAD4 cannot substitute GR for *Pparg2* induction (Fig. 5c–e). Interestingly, overexpression of GR reduced the amount of C/EBPβ protein, which limited the MDI-mediated induction of PPARγ (Fig. 5f, g). This finding is consistent with previous study, which showed that liganded GR inhibits cAMP-activated CREB, an important transcription factor required for the induction of *Cebpb*^43^. We overexpressed GR together with C/EBPβ in 3T3-L1 cells (GR/Cβ-L1 cells). Unlike the ectopic expression of only C/EBPβ (Cβ-L1 cells), overexpression of both GR and C/EBPβ resulted in reinitiation of adipogenesis in 3T3-L1 cells even in the presence of Wnt3a (Fig. 5h). Although Wnt3a still reduced early induction of PPARγ, GR/Cβ-L1 cells maintained sufficiently high PPARγ protein level to enable completion of adipogenesis even in the presence of Wnt3a (Fig. 5h, i). ChIP analyses also showed that in GR/Cβ-L1 cells, Wnt3a did not reduce GR bindings to the *Pparg2* promoter (Fig. 5j). These results corroborated the observation that GR protein level remained high in the nuclei of GR/Cβ-L1 cells even in the presence of Wnt3a (Fig. 5k) and demonstrated that Wnt3a blocked MDI-mediated induction of both *Tead4* and *Pparg2* by limiting GR in nuclei during early adipogenesis.

### β-Catenin is necessary for conveying the Wnt3a signal to the Pparg2 promoter

**Fig. 6:**
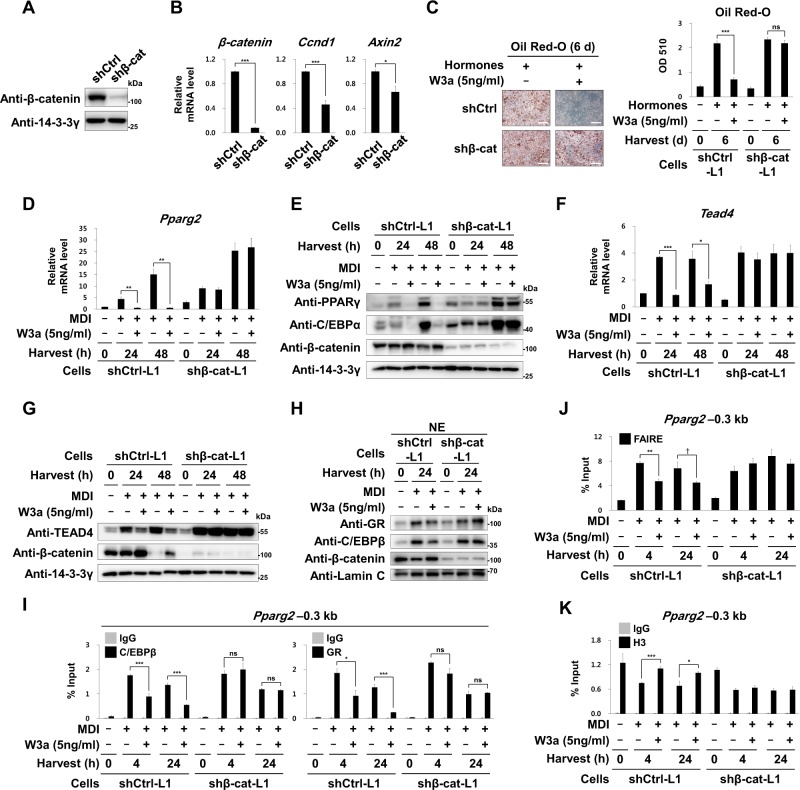
Effects of β-catenin knockdown. **a**–**k** 3T3-L1 preadipocytes were infected with a lentivirus encoding shRNA against mouse *β-catenin* (shβ-cat-L1 cells) or control shRNA (shCtrl-L1 cells). **a** Western blot analyses showing β-catenin level. **b** qRT-PCR analyses showing relative mRNA levels of *β-catenin*, *Ccnd1*, and *Axin2* to 18S rRNA levels. **c**–**k** The shCtrl-L1 or shβ-cat-L1 cells were induced to undergo adipogenesis by treating with adipogenic hormones for the indicated time points in the presence or absence of W3a (5 ng/ml). **c** Images and optical densities (510 nm) of Oil Red-O stained lipid. Scale bars, 200 μm. **d**, **f** Relative mRNA levels of *Pparg2* and *Tead4* to 18S rRNA levels. **e**, **g** Western blot analyses using the indicated antibodies. **h** Western blot analyses of nuclear extracts (NE) of the shCtrl-L1 or shβ-cat-L1 cells using the indicated antibodies. Lamin C was used as the loading control for nuclear proteins. **i**, **k** ChIP-qPCR analyses of C/EBPβ, GR, or H3 occupancy on the –0.3 kb region from TSS of *Pparg2*. **j** FAIRE-qPCR analyses on the –0.3 kb region from TSS of *Pparg2*. qPCR data show mean ± SE. All data were repeated at least three independent same or similar experiments. *p < 0.05, **p < 0.01, and ***p < 0.001, † *p* *=* 0.065 by Students’ *t*-test; ns, not significant

Knockdown of β-catenin reduced the mRNA levels of its target genes such as *Axin2* and *Ccnd1* in 3T3-L1 cells (Fig. 6a, b) and completely blocked the inhibitory effects of Wnt3a on adipogenesis and induction of both *Pparg2* and *Tead4* (Fig. 6c–g). We found that Wnt3a did not reduce the nuclear amounts of GR proteins in shβ-cat-L1 cells (Fig. 6h). Consequently, in 3T3-L1 cells with β-catenin knockdown, Wnt3a did not block the occupancies of C/EBPβ and GR on the *Pparg2* promoter (Fig. 6i) and did not inhibit the opening of chromatin structure (Fig. 6j, k). These results indicate that β-catenin is necessary for conveying the Wnt3a signal to the chromatin of *Tead4* and *Pparg2* by limiting the nuclear localization of GR.

### Wnt3a blocks remodeling of actin cytoskeleton in a β-catenin-dependent manner

**Fig. 7:**
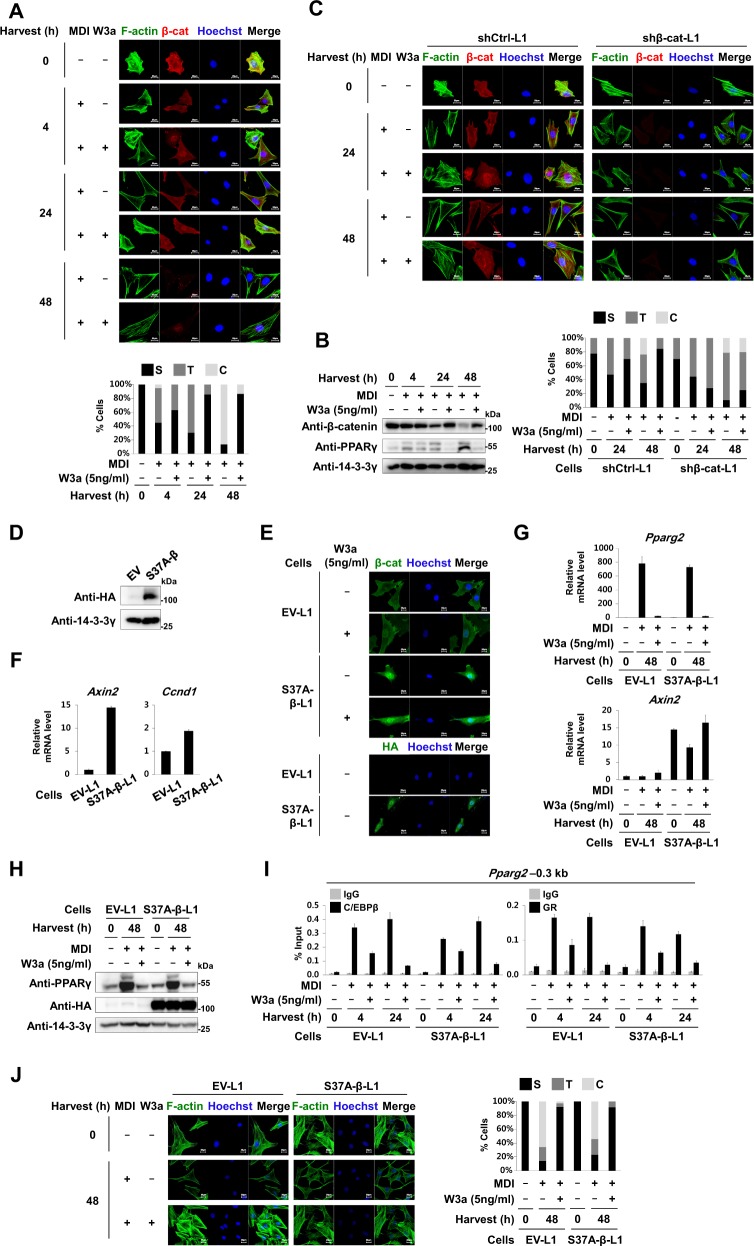
Cytoskeletal rearrangement during early adipogenesis. 3T3-L1 cells were treated with MDI for the indicated time points in the presence or absence of W3a (5 ng/ml) as described in Fig. 1a. **a** Confocal microscopic images of cellular filamentous actin (F-actin) and β-catenin (upper panel). F-actin structures in individual cells were categorized into three groups. S (stress fiber), where F-actin stress fibers were observed in both nuclei and cytoplasm; T (transition status), where F-actin stress fibers were observed in the cytoplasm but not in the nucleus; C (cortical structure), where F-actin stress fibers were observed neither in the nucleus nor in the cytoplasm, but F-actin was observed near the cellular membrane. Cells (13–48) in each treatment were observed and categorized into three groups. The graph indicates the percentage of cells in each category (lower panel). **b** Western blot analyses of 3T3-L1 cells using the indicated antibodies. **c** Confocal microscopic images of cellular F-actin and β-catenin (upper panel). The graph indicates the percentage of cells in each category (S, T, and C described in Fig. 7a) (lower panel). **d**–**j** 3T3-L1 preadipocytes were infected with retrovirus encoding HA-tagged S37A-β-catenin (S37A-β-L1 cells) or empty vector (EV-L1 cells) as a control. **d** Western blot analyses of the EV-L1 cells or the S37A-β-L1 cells using anti-HA and 14-3-3γ antibodies. **e** Confocal microscopic images of the EV-L1 cells or the S37A-β-L1 cells immunostained with either anti-β-catenin antibody or anti-HA antibody. The cells were treated with W3a (5 ng/ml) for 24 h. The nuclei were stained with Hoechst 33258 (blue). **f** qRT-PCR analyses showing relative mRNA levels of *Axin2* and *Ccnd1* to 18S rRNA. **g**–**j** The EV-L1 cells or the S37A-β-L1 cells were treated with MDI for the indicated time points in the presence or absence of W3a (5 ng/ml). **g** Relative mRNA levels of *Pparg2* and *Axin2* to 18S rRNA. **h** Western blot analyses using the indicated antibodies. **i** ChIP-qPCR analyses of C/EBPβ or GR occupancy on the –0.3 kb region from TSS of *Pparg2*. **j** Confocal microscopic images of the cells immunostained with fluorescent phalloidin conjugates (green) and with Hoechst 33258 (blue) (left panel). Graph indicating the percentage of cells in each category (S, T, and C described in Fig. 7a) (right panel). qPCR data show mean ± SE. All data were repeated at least three independent same or similar experiments

β-Catenin is connected to the actin cytoskeleton via interactions with α-catenin and cadherin proteins in cytoplasm^26^. Upon exposure to adipogenic hormones, 3T3-L1 cells differentiate into round and lipid-laden adipocytes, accompanied by changes in the actin cytoskeleton from stress fibers to cortical structures^4^. Time course staining with fluorescent phalloidin, a probe specific for filamentous actin (F-actin), revealed that MDI reduced F-actin stress fibers in 50% cells from 4 h, whereas they disappeared in 86% cells within 48 h; instead, F-actin reorganized only at the cortical region. The amount of β-catenin protein decreased during the rearrangement of F-actin from stress fibers to cortical structures. Wnt3a prevented MDI from reducing β-catenin protein and disrupting F-actin stress fibers (Fig. 7a, b). However, this was not observed in shβ-cat-L1 cells, suggesting that β-catenin is required for Wnt3a to prevent F-actin rearrangement (Fig. 7c). However, we observed that active S37A-β-catenin which is constitutively present in the nuclei, is not sufficient to elicit the inhibitory effects of Wnt3a on remodeling of chromatin and cytoskeleton during early adipogenesis although at late phase of adipogenesis, ectopic expression of S37A-β-catenin can reduce the mRNA and protein levels of *Pparg2* even in the absence of Wnt3a (Fig. 7d–j and S5A to S5C).

**Fig. 8:**
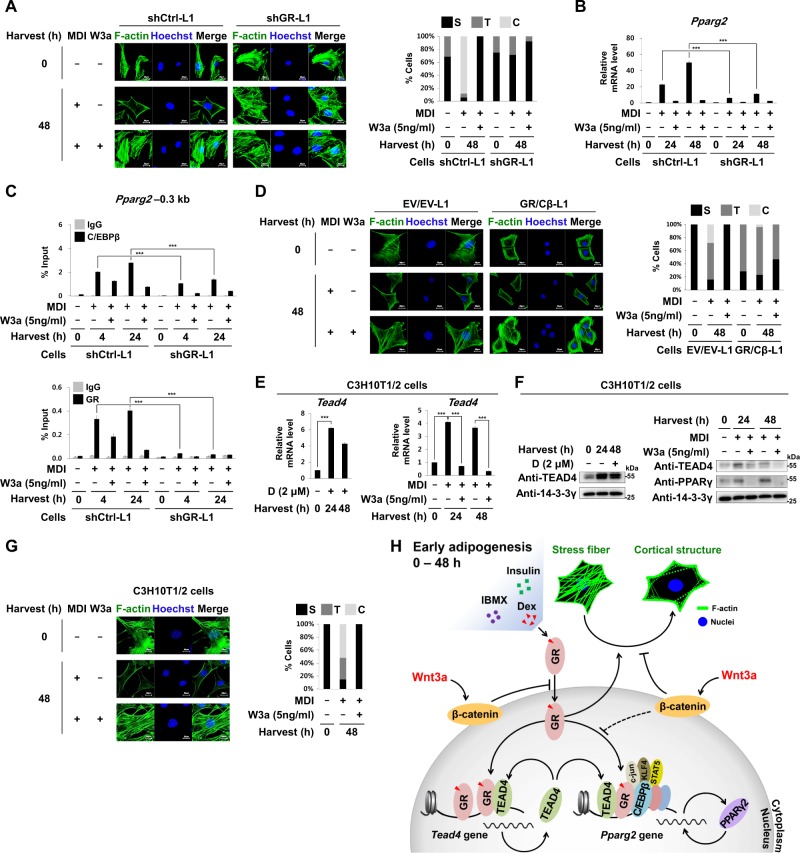
Effects of GR on cytoskeletal rearrangement. The shCtrl-L1 or shGR-L1 cells were induced to undergo adipogenesis by treating with adipogenic hormones for the indicated time points in the presence or absence of W3a (5 ng/ml). **a** Confocal microscopic images of F-actin stress fibers (left panel). The graph indicates the percentage of cells in each category (S, T, and C described in Fig. 7a) (right panel). **b** qRT-PCR analyses of *Pparg2* mRNA levels to 18S rRNA levels. **c** ChIP-qPCR analyses of C/EBPβ or GR occupancy on the –0.3 kb region from TSS of *Pparg2*. **d** Confocal microscopic images of cellular F-actin stress fibers (left panel) in the EV/EV-L1 or GR/Cβ-L1 cells. The graph indicates the percentage of cells in each category (S, T, and C described in Fig. 7a) (right panel). **e**–**g** C3H10T1/2 cells were treated with Dex (2 μM) or MDI for the indicated time points in the presence or absence of W3a (5 ng/ml). **e** qRT-PCR analyses of *Tead4* and *Pparg2* mRNA levels to 18S rRNA levels. **f** Western blot analyses showing TEAD4 and PPARγ protein levels. **g** Confocal microscopic images of cellular F-actin stress fibers (left panel) in C3H10T1/2 cells. The graph indicates the percentage of cells in each category (S, T, and C described in Fig. 7a) (right panel). **h** Schematic diagram showing the inhibitory effects of Wnt3a/β-catenin on positive circuits of GR-TEAD4-PPARγ2 and cytoskeletal remodeling during early adipogenesis (details in Results). qPCR data show mean ± SE. All data were repeated at least three independent same or similar experiments. ***p < 0.001 by Students' *t*-tests

As Wnt3a reduced nuclear GR protein level in a β-catenin-dependent manner (Fig. 6h), we investigated whether GR is necessary for MDI to rearrange F-actin. In shGR-L1 cells with GR knockdown, MDI could neither rearrange F-actin nor induce the mRNA level of *Pparg2* (Fig. 8a, b). GR knockdown reduced MDI-induced binding of C/EBPβ to the *Pparg2* promoter (Fig. 8c). In contrast, in GR/Cβ-L1 cells without MDI treatment, F-actin stress fibers were observed only around the peripheral region but not in the nuclei of 3T3-L1 cells (Fig. 8d). Wnt3a did not recover F-actin stress fibers in GR/Cβ-L1 cells. Like 3T3-L1 cells, in C3H10T1/2, mouse mesenchymal stem cells, both Dex and MDI increased the mRNA and protein levels of TEAD4 and PPARγ which were also inhibited by Wnt3a (Fig. 8e, f). Furthermore, Wnt3a prevented MDI from rearranging F-actin stress fibers in C3H10T1/2 cells (Fig. 8g). These findings suggest that during early adipogenesis, GR is necessary both for the rearrangement of F-actin and hotspot formation on the *Pparg2* promoter, and that Wnt3a blocks these two events by limiting the nuclear level of GR in a β-catenin-dependent manner (Fig. 8h).

## Discussion

The findings that treatment of Dex followed by IBMX was sufficient for adipogenesis, but that IBMX treatment followed by Dex treatment did not recapitulate this effect suggested that Dex primed preadipocytes to a novel commitment state for adipogenesis^44^. Adipogenesis of mouse embryonic fibroblasts (MEFs) isolated from *GR* (*Nr3c1*) knockout and GR dimerization-defective mutant mice was impaired^45^. Knockdown of GR or omission of Dex from MDI did not induce *Pparg2* in 3T3-L1 cells^8,46^. These previous studies indicated that GR is essential for *Pparg2* induction.

Using extensive genome-wide profiling of 15 TFs at 4 h after MDI treatment, Mandrup and colleagues demonstrated that during early adipogenesis, hotspots co-occupied by more TFs recruited more coactivators such as p300/CBP to constitute super-enhancers^1^. Furthermore, enhancers with large numbers of TFs are more sensitive to small changes in TF concentration compared to those with smaller numbers of TFs^47–49^. This is consistent with our findings that reduction in nuclear GR level by β-catenin sensitively changes super-enhancer formation on *Pparg2* and other genes (Fig. S2B). Our findings that GR amplifies MDI signals to *Pparg2* by inducing TEAD4, a novel TF for *Pparg2*, highlighted that changes in GR activity affect *Pparg2* expression (Fig. 3).

ChIP-exo sequencing using GR antibody in IMR90 cells revealed that TEAD4 and GR co-occupied GR target genes as a heterodimer and that TEAD knockdown decreased the expression of several GR target genes^42^. Biochemical CAP-SELEX analyses which identify cooperative interactions between TF pairs and heterodimeric DNA motifs, revealed that TEAD4 is the most common partner that cooperatively binds diverse DNA sequences with 32 TFs among 100 tested TFs. TEAD4 and its partner TFs recognize composite sequences that were considerably different from the individual TF motifs, suggesting that in vivo functions of TEAD4 may encompass diverse biological functions depending on its partner TFs^50^. This is the first study to show that TEAD4 cooperatively binds to *Pparg2* together with other hotspot TFs, and that Tead4 knockdown reduced *Pparg2* expression. Since GR and TEAD4 cooperatively bind and induce *Tead4*, GR is a key TF that persistently drives expression of both *Tead4* and *Pparg2*. Wnt3a disrupted two mutually related positive circuits by limiting GR binding to the promoters of these two genes.

Goentoro and Kirschner have showed that fold-change, but not the absolute level of β-catenin, regulates Wnt signaling in an experimental model of *Xenopus* dorsal-anterior development^51^. Our findings that the nuclear form of S37A-β-catenin failed to deliver Wnt3a signal imply that Wnt3a disrupts hotspot formation and cytoskeletal rearrangement but not by increasing the coactivator activity of β-catenin in the nuclei (Fig. 7d–j). Similar to β-catenin, the TAZ coactivator activated by the canonical Wnt pathway, inhibits adipogenesis by inhibiting PPARγ protein^52,53^. We found that the protein level of TAZ decreased during early adipogenesis, but not in the presence of Wnt3a, suggesting that TAZ can be a mediator that delivers the Wnt signal to *Pparg2*. However, our finding that TAZ knockdown did not block Wnt3a inhibitory effects indicates that TAZ is not essential for the anti-adipogenic function of Wnt3a (Fig. 4k).

Unlike lipogenesis in other cells such as hepatocytes and myocytes, adipogenesis requires dramatic cytoskeletal remodeling of F-actin stress fibers to cortical actin structures, which is required to hold a large lipid vacuole in the center, relocate the nucleus and other organelles, and attain a round shape. Cytoskeletal remodeling starts within 24 h after adipogenic hormone treatments^13^, suggesting that cytoskeletal remodeling is followed by complete induction of lipogenic genes as a feed-forward mechanism. These nuclear and cytoplasmic events should interdependently regulate each other to prevent metabolic and structural catastrophes during adipogenesis^54,55^. In addition to adipogenic hormones, the stiffness of extracellular matrices (ECM) is involved in commitment for adipogenesis. Primary preadipocytes embedded in stiffer matrices showed reduced rates of adipogenesis^10^. ECM stiffness increases tissue tension, which leads to increased actin and myosin fiber and cell stretching^56,57^. Interestingly, mesenchymal stem cells exposed to mechanical strain show increase in β-catenin level and could not differentiate into adipocytes, suggesting that Wnt/β-catenin conveys signals from intracellular tension to the nuclei^58^. In agreement with this result, we found that Wnt signal could not prevent MDI from changing F-actin stress fibers to cortical F-actin structures during adipogenesis in the absence of β-catenin.

Interestingly, Wnt3a reduced GR level in the nuclei during the early phase of adipogenesis, but not in Dex-treated preadipocytes, suggesting that the antagonistic effect of Wnt/β-catenin on GR is specific for adipogenesis (Figs. 1i, j,  6h). Extensive studies have revealed that the chaperone complex and intact cytoskeleton are required for the nuclear transport of GR^59^. Furthermore, GR is connected to actin filaments through HSP90, a main component of GR-chaperone complex suggesting that the nuclear transport of GR and cytoskeletal rearrangement are closely related^60^. Upon ligand binding, the GR-chaperone complex recruits a motor protein dynein to form the liganded GR-HSP90-FKBP52-dynein complex, which is able to move along the microtubules through the nuclear pore complex (NPC) to the nucleus^61,62^. However, when the cytoskeleton is disrupted like adipogenesis, liganded GR simply diffuses in and out of the nucleus mainly via importins, exportins, and the RanGTPase system^59,63^. The mechanism via which the liganded-GR translocates into the nucleus during adipogenic cytoskeletal rearrangement remains unknown. In contrast, β-catenin harbors armadillo repeats, which are similar to the importin-β HEAT repeats^64–66^. Therefore, β-catenin can pass through NPC like importin and equilibrates between the nucleus and the cytoplasm by passive diffusion. Therefore subcellular distribution of β-catenin in the nuclei, cytoplasm, and the membrane can be determined from its location and the levels of diverse interacting complexes, namely, the TCF/LEF transcription factors in the nucleus, APC and AXIN in the cytoplasm, and cadherin complex in the membrane^67^. It remains to be investigated whether GR and β-catenin compete with each other for nuclear translocation during adipogenic cytoskeletal rearrangement.

In conclusion, this study provides insights into an intriguing question regarding chromatin remodeling and actin rearrangement, which are interdependently regulated during adipogenesis. Our findings that GR is necessary for the rearrangement of both cytoskeleton and chromatin, and that canonical Wnt3a inhibited both processes in a β-catenin-dependent manner, suggest that rearrangements of both chromatin and cytoskeleton are related in ways that involve the antagonistic activities of GR and β-catenin, and that Wnt3a reinforced β-catenin function.

## Supplementary information

Clean_Supplementary_Figures

Supplementary_Tables
