# Supplementary material for: Wnt3a disrupts GR-TEAD4-PPARγ2 positive circuits and cytoskeletal rearrangement in a β-catenin-dependent manner during early adipogenesis
Source: Cell Death Dis. 2019 Jan 8;10(1):16. doi: 10.1038/s41419-018-1249-7 (PMC6325140; doi:10.1038/s41419-018-1249-7)
Supplement: Supplementary file 1 — Clean_Supplementary_Figures [file 41419_2018_1249_MOESM1_ESM.docx]

**Supplementary FIGUREs**

**Wnt3a disrupts GR-TEAD4-PPARγ2 positive circuits and cytoskeletal rearrangement in a β-catenin-dependent manner during early adipogenesis**

Bongju Park ^1^, Soojeong Chang ^1^, Gwan-Jun Lee ^1^, Byeongsoo Kang ^2^, Jong Kyoung Kim ^3^ and Hyunsung Park ^1*^

^1^ Department of Life Science, University of Seoul, Seoul 02504, Republic of Korea, ^2^ SYSOFT R&D CENTER, Daegu 42988, Republic of Korea, ^3^ Department of New Biology, Daegu Gyeongbuk Institute of Science and Technology, Daegu 42988, Republic of Korea

**Supplementary Figure S1**


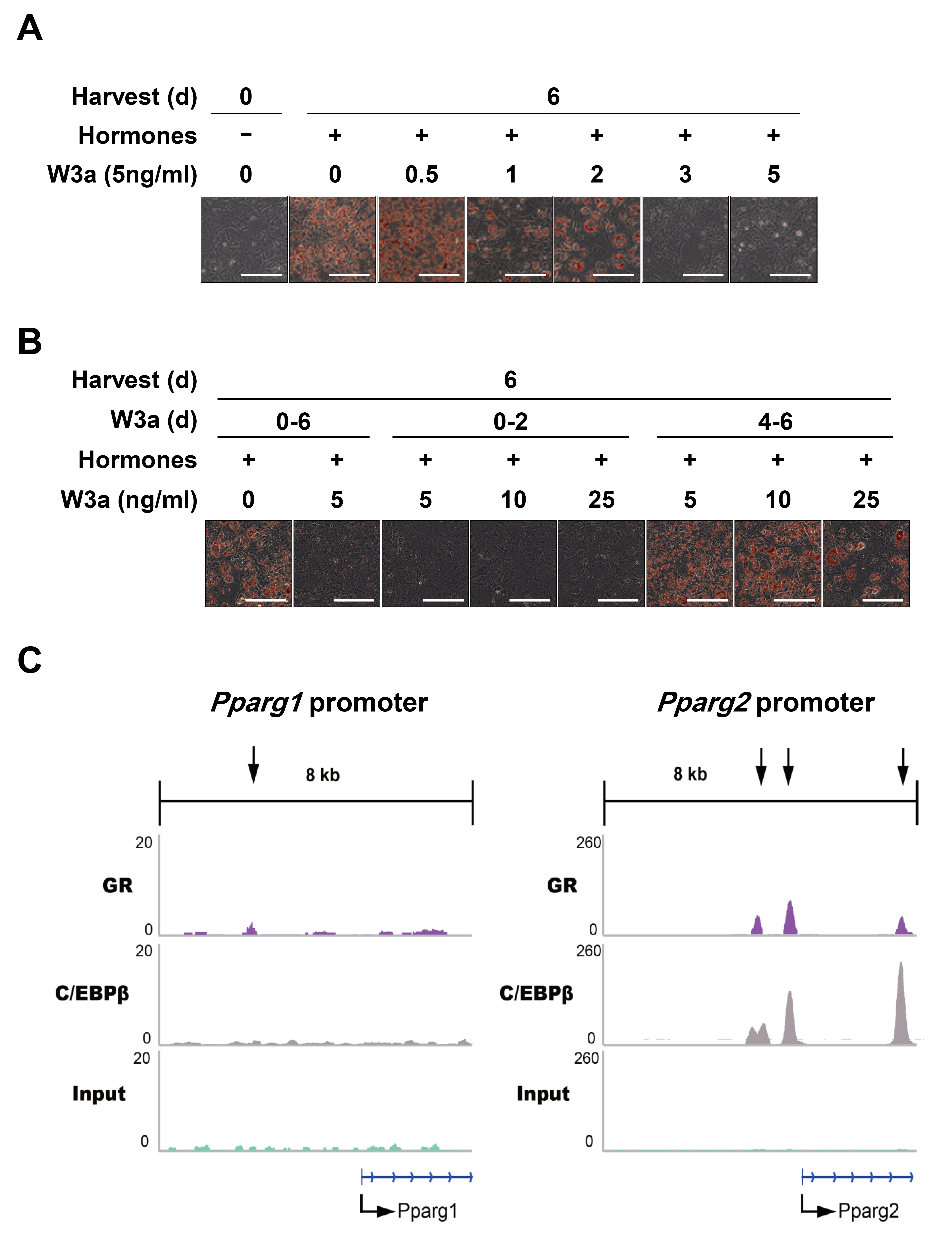


**Supplementary Figure S1. Effective dose of Wnt3a, and *Pparg* gene at early adipogenesis** (A-B) Images of Oil Red-O stained lipid in 3T3-L1 cells that were treated with adipogenic hormones for six days in the presence of the indicated doses of recombinant mouse Wnt3a (details in Methods). Scale bars, 200 μm. (C) Binding profiles of GR or C/EBPβ in the promoters (–5 kb to +3 kb of TSS) of *Pparg1* and *Pparg2* genes that were selected from publicly available ChIP-seq dataset (GSE27826) of 3T3-L1 cells treated with MDI for 4 h. Histogram represents GR or C/EBPβ binding peaks on the promoter of the indicated genes at 4 h of MDI treatment.

**Supplementary Figure S2**





**Supplementary Figure S2 continued**


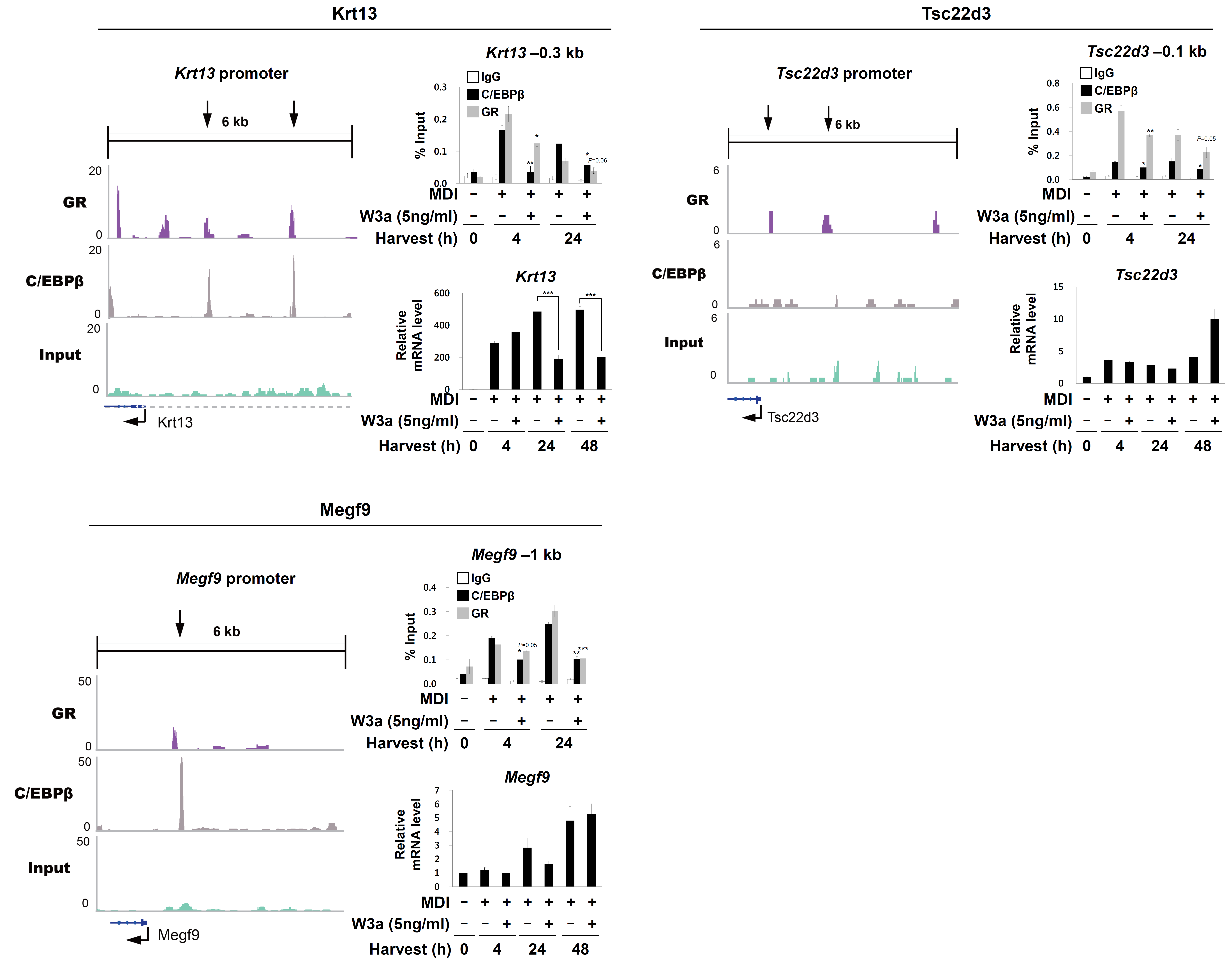


**Supplementary Figure S2. Effects of Wnt3a on GR** (A) Western analyses of nuclear extracts (NE) of 3T3-L1 cells treated with adipogenic hormones for 4 h in the presence or absence of W3a (5 ng/ml). Lamin C was used as the loading control for nuclear proteins. The relative band intensities of GR, C/EBPβ, and lamin C were determined using the ImageJ software. Values represent mean and S.E. obtained from four independent experiments. (B) Binding profiles of GR or C/EBPβ in the promoters (–5 kb to +1 kb of TSS) of seven MDI-induced genes that were selected from publicly available ChIP-seq dataset (GSE27826) of 3T3-L1 cells treated with MDI for 4 h. Single-end reads of ChIP-seq data were mapped to the *Mus musculus* genome (mm10) using Bowtie2 (version 2.2.8). Peak calling was performed by HOMER (version 4.9.1) and the input was used as a control. The peaks were mapped to nearby genes using HOMER annotatePeaks.pl. Each ChIP-seq experiment was normalized to contain 10 million tags and the normalized signal of ChIP-seq occupancy was visualized with IGV (version 2.4). Histogram represents GR or C/EBPβ binding peaks on the promoter of the indicated genes at 4 h of MDI treatment (left panel of each gene). ChIP-qPCR analyses of GR or C/EBPβ occupancy and qRT-PCR analyses of relative mRNA levels of the indicated genes were performed using 3T3-L1 cells at the indicated time points of differentiation (right panel of each gene). Values obtained from ChIP-qPCR and qRT-PCR analyses present the mean ± S.E. of two independent experiments. **p* < 0.05, ***p* < 0.01, and ****p* < 0.001 by Students’ *t*-test.

**Supplementary Figure S3**


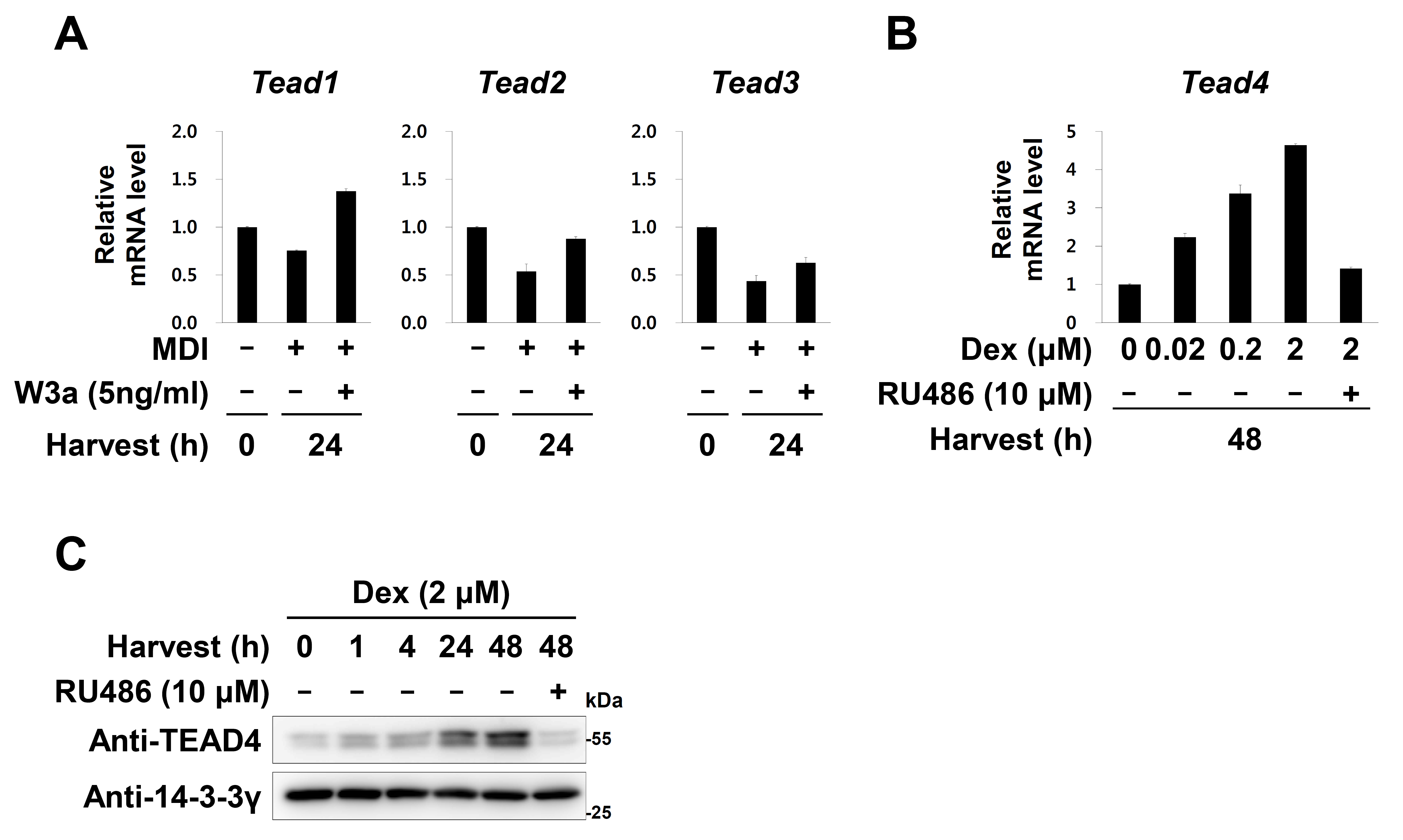


**Supplementary Figure S3. GR-dependent induction of *Tead4*** (A) Relative mRNA levels of *Tead1*, *Tead2,* and *Tead3* to 18S rRNA in 3T3-L1 cells treated with MDI (24 h) in the presence or absence of W3a (5 ng/ml). (B and C) 3T3-L1 cells were treated with indicated doses of Dex or RU486 (10 μΜ) for the indicated time points. (B) qRT-PCR analyses of *Tead4* mRNA levels. (C) Western blot analyses using anti-TEAD4 and 14-3-3γ antibodies. 14-3-3γ was used as the loading control.

**Supplementary Figure S4**


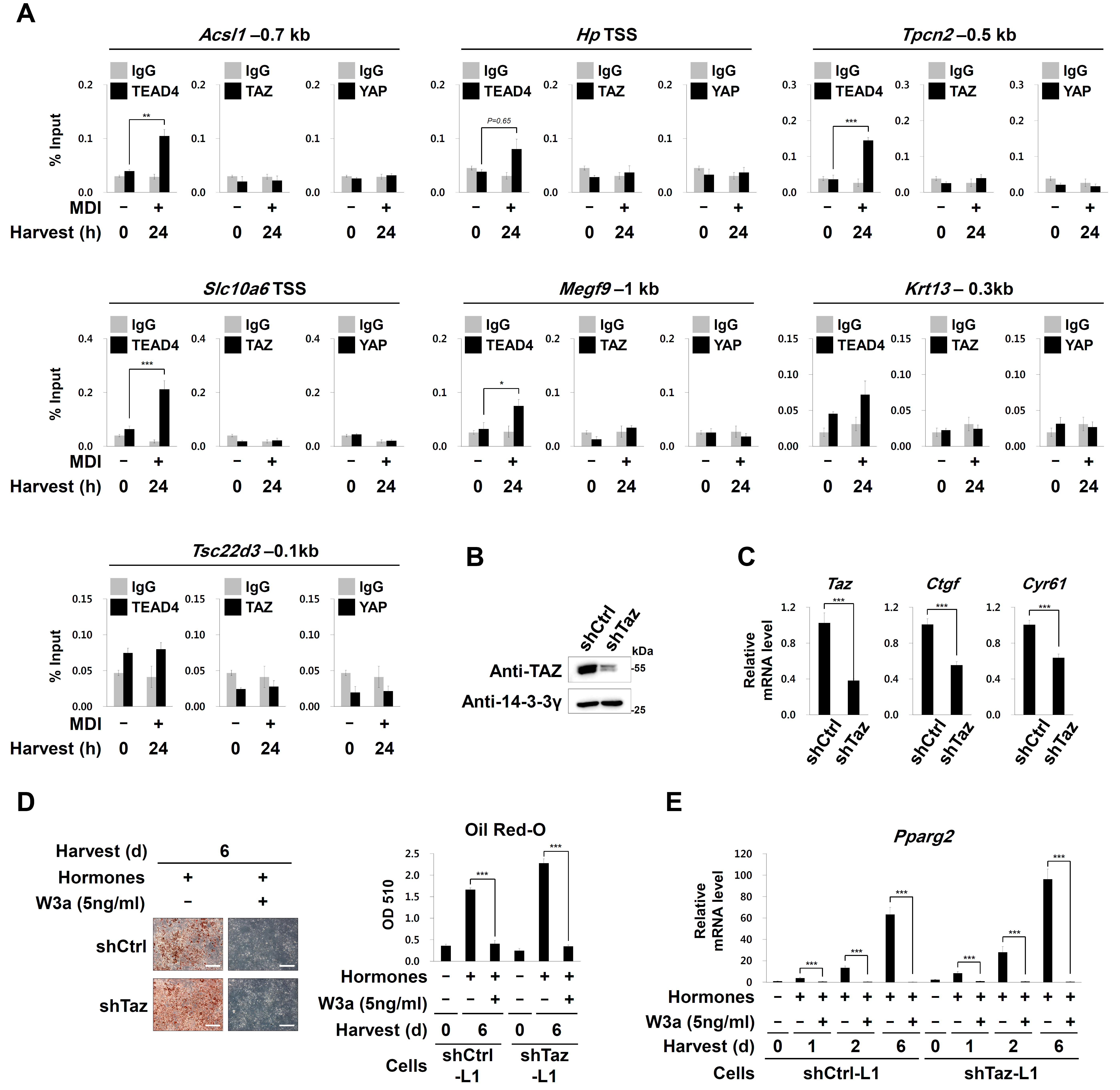


**Supplementary Figure S4. TEAD4, TAZ, and YAP during early adipogenesis** (A) ChIP-qPCR analyses of TEAD4, TAZ or YAP bindings on the promoter of seven MDI-induced genes which were described n in Figure S2B. (B-E) 3T3-L1 preadipocytes were infected with lentiviruses encoding shRNA against mouse Taz (5’-CCTGCATTTCTGTGGCAGATA-3’) (shTaz-L1 cells) or control shRNA (shCtrl-L1 cells). (B) Western blot analyses showing TAZ protein levels. (C) qRT-PCR analyses showing the relative levels of Taz, Ctgf, and Cyr61 mRNA to 18S rRNA. Ctgf and Cyr61 are target genes of TAZ. (D, E) The shCtrl-L1 or shTaz-L1 cells were treated with adipogenic hormones for the indicated time points in the presence or absence of W3a (5 ng/ml). (D) Images and optical densities (510 nm) of Oil Red-O stained lipid. Scale bars, 200 μm. (E) Relative levels of Pparg2 mRNA to 18S rRNA. Data show the mean ± S.E. of two (B, D) or three (A, C) independent experiments. *p < 0.05, **p < 0.01, and ***p < 0.001 by Students’ t-test.

**Supplementary Figure S5**


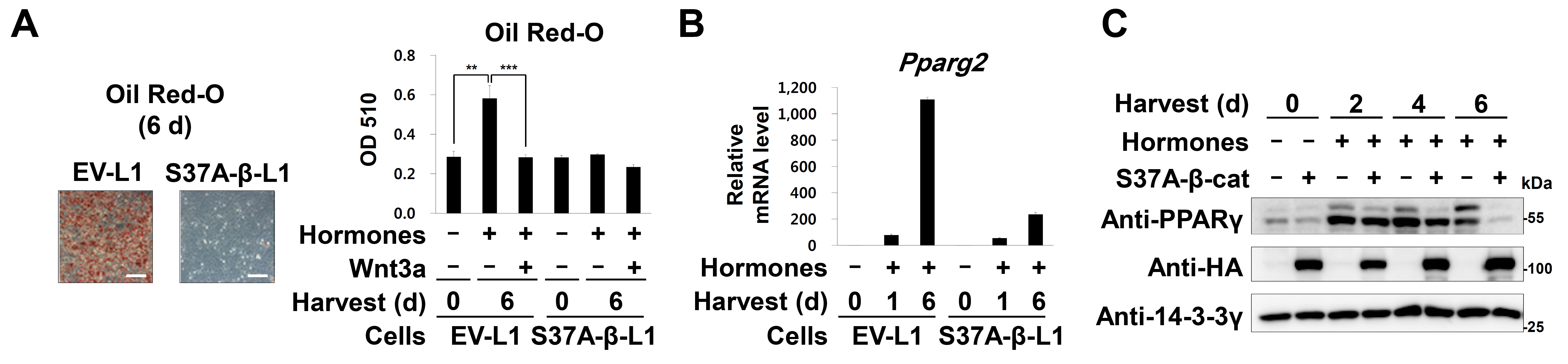


**Supplementary Figure S5. Effects of S37A-β-catenin on late adipogenesis** (A) Images and optical densities (510 nm) of Oil Red-O stained lipid in the EV-L1 cells or the S37A-β-L1 cells that were treated with adipogenic hormones for six days. Scale bars, 200 μm. (B) qRT-PCR analyses showing the relative levels of *Pparg2* mRNA to 18S rRNA in the EV-L1 cells or the S37A-β-L1 cells at the indicated days after exposure to adipogenic hormones. (C) Western blot analyses using the indicated antibodies. 14-3-3γ was used as the loading control.
