# Supplementary material for: Wnt3a disrupts GR-TEAD4-PPARγ2 positive circuits and cytoskeletal rearrangement in a β-catenin-dependent manner during early adipogenesis
Source: Cell Death Dis. 2019 Jan 8;10(1):16. doi: 10.1038/s41419-018-1249-7 (PMC6325140; doi:10.1038/s41419-018-1249-7)
Supplement: Supplementary file 2 — Supplementary_Tables [file 41419_2018_1249_MOESM2_ESM.docx]

**Supplementary TABLES**

**Supplementary Table S1. Primer sequences used in qRT-PCR analyses**.

| **Mouse Gene** | **Strand** | **Sequence** |
| --- | --- | --- |
| *18S rRNA* | Sense | ACCGCAGCTAGGAATAATGGAATA |
|  | Antisense | CTTTCGCTCTGGTCCGTCTT |
| *Acsl1* | Sense | TGCCAGAGCTGATTGACATTC |
|  | Antisense | GGCATACCAGAAGGTGGTGAG |
| *Axin2* | Sense | ATGGAGTCCCTCCTTACCGCAT |
|  | Antisense | GTTCCACAGGCGTCATCTCCTT |
| *β-catenin* | Sense | GTTCGCCTTCATTATGGACTGCC |
|  | Antisense | ATAGCACCCTGTTCCCGCAAA |
| *Cebpa* | Sense | GAACAGCAACGAGTACCGGGTA |
|  | Antisense | GCCATGGCCTTGACCAAGGAG |
| *Ctgf* | Sense | CTGCCTACCGACTCGAAGAC |
|  | Antisense | CATTGGTAACTCGGGTGGAG |
| *Ccnd1* | Sense | GCTGCAAATGGAACTGCTTC |
|  | Antisense | AGGGTGGGTTGGAAATGAAC |
| *Cyr61* | Sense | GCTCAGTCAGAAGGCAGACC |
|  | Antisense | GTTCTTGGGGACACAGAGGA |
| *Hp* | Sense | GCTATGTGGAGCACTTGGTTC |
|  | Antisense | CACCCATTGCTTCTCGTCGTT |
| *Krt13* | Sense | GACTGGCATCTGAAACAGAGCC |
|  | Antisense | TTGTCCGTGGTGGCTTCCAGAA |
| *Megf9* | Sense | AGCAGCCCCTTCCCTAAGA |
|  | Antisense | AGTGTGTCCGTCCTCCGAA |
| *Pparg2* | Sense | CTGATGCACTGCCTATGAGCA |
|  | Antisense | ATGCGAGTGGTCTTCCATCAC |
| *Slc10a6* | Sense | TGCAACCAGAGCTTCTGACTC |
|  | Antisense | GGAGGGCCATGCGAATCTAAA |
| *Taz* | Sense | GTCACCAACAGTAGCTCAGATCC |
|  | Antisense | GTTGCTGAGGAAGTCTTCTGGAG |
| *Tead1* | Sense | TCCGCTTTCCTTGAACAGCAGAG |
|  | Antisense | GGGTCACTGTAAGAATGGTTGGC |
| *Tead2* | Sense | AAGGTCTGCTCCTTTGGCAAGC |
|  | Antisense | CTGACGGAGCTTGTGCAGGAAA |
| *Tead3* | Sense | ATCGTCTCTGCCAGCGTTCTAC |
|  | Antisense | CTGAGAAGGTCCAGGCTGTTGT |
| *Tead4* | Sense | GCTCTGGATGTTGGAGTTCTCG |
|  | Antisense | TTGGGCTTGACTGGCTGATGTG |
| *Tpcn2* | Sense | CACGACTGATGAACACACTGA |
|  | Antisense | CCAGGAGGCACGATGACAC |
| *Tsc22d3* | Sense | ACCACCTGATGTACGCTGTG |
|  | Antisense | CAGCCGGGACTGGAACTTT |

**Supplementary Table S2. Primer sequences used in ChIP-qPCR analyses**.

| **PCR Region** | **Strand** | **Sequence** |
| --- | --- | --- |
| *Pparg2* –0.3kb | Sense | ACCGCAGCTAGGAATAATGGAATA |
|  | Antisense | CTTTCGCTCTGGTCCGTCTT |
| *Pparg2* + 2.6kb | Sense | TGCCAGAGCTGATTGACATTC |
|  | Antisense | GGCATACCAGAAGGTGGTGAG |
| *Tead4* –0.9kb | Sense | GGGTCTGTTTCTGCACTGAGC |
|  | Antisense | AGCAAGCGTATCTGTGAGCGT |
| *Tead4* + 0.3kb | Sense | GGTCTCCTCCCAAAGGTG |
|  | Antisense | GACTGGAGTTTGCAGAGGA |
| *Acsl1* –0.7kb | Sense | TGGACACCGTCCTTGAGCA |
|  | Antisense | CGCAATATCCTGGCCATGAG |
| *Ctgf* –0.1kb | Sense | CTTCTTGGTGTTGTGCTGGA |
|  | Antisense | GATTGATCCTGACCCCTTGA |
| *Hp* TSS | Sense | ACACAACGCAGAGGGCCAA |
|  | Antisense | CGTCTCTAAGGTCACTGGCTG |
| *Krt13* –0.3kb | Sense | GAGAGTCTCCGCTACTGAA |
|  | Antisense | AAGACTTGGCAGGAGTCATG |
| *Megf9* –1kb | Sense | GCAATGCGGGCTGCTTTC |
|  | Antisense | ATGCTTGGCCCACGGTTG |
| *Slc10a6* TSS | Sense | TCTCATTCCCAGCATTGAACG |
|  | Antisense | AAGTAATGTGCTTGGGCAGGA |
| *Tpcn2* –0.5kb | Sense | CACATGCGTCAGAGTCACGC |
|  | Antisense | AGGCAAGCTCTCAACCCCTG |
| *Tsc22d3* –0.1kb | Sense | GCCCTCTGCTGAATGCTTTC |
|  | Antisense | AAGAGGACTTTGGAGGCTGG |
